# Immune Repertoire Profiling Reveals Distinct Adaptive Immune Signatures of Dampness ZHENG Across Psoriasis, Rheumatoid Arthritis and Ulcerative Colitis

**DOI:** 10.1111/cpr.70265

**Published:** 2026-07-18

**Authors:** Lipeng Tang, Maojie Wang, Yuhong Yan, Shumin Qin, Youbang Liang, Danni Yao, Liyan Mei, Hao Deng, Shuyan Ye, Xiumin Chen, Kaixin Gao, Xirun Zheng, Taohua Liu, Xinmin Qiu, Song Li, Xinjie Yu, Wenjing Pan, Shaogang Huang, Zhe Wang, Runyue Huang, Guangjuan Zheng, Chuanjian Lu

**Affiliations:** ^1^ State Key Laboratory of Dampness Syndrome of Chinese Medicine The Second Clinical College of Guangzhou University of Chinese Medicine Guangzhou China; ^2^ Guangdong‐Hong Kong‐Macau Joint Lab on Chinese Medicine and Immune Disease Research The Second Clinical College of Guangzhou University of Chinese Medicine Guangzhou China; ^3^ Department of Pharmacology of Traditional Chinese Medicine The Second Clinical College of Guangzhou University of Chinese Medicine Guangzhou China; ^4^ Department of Rheumatology Clinical and Basic Research The Second Clinical College of Guangzhou University of Chinese Medicine Guangzhou China; ^5^ Department of Dermatology The Second Clinical College of Guangzhou University of Chinese Medicine Guangzhou China; ^6^ Department of Gastroenterology The Second Clinical College of Guangzhou University of Chinese Medicine Guangzhou China; ^7^ Department of Pathology The Second Clinical College of Guangzhou University of Chinese Medicine Guangzhou China; ^8^ Genetic Testing Lab The Second Clinical College of Guangzhou University of Chinese Medicine Guangzhou China; ^9^ Hengyang Medical School University of South China Hengyang Hunan China; ^10^ National Health Commission Key Laboratory of Birth Defect Research and Prevention Hunan Provincial Maternal and Child Health Care Hospital Changsha Hunan China; ^11^ Institute for Future Sciences, University of South China Changsha Hunan China; ^12^ Department of Gastroenterology The First Affiliated Hospital of Guangzhou University of Chinese Medicine Guangzhou China; ^13^ State Key Laboratory of Traditional Chinese Medicine Syndrome The Second Affiliated Hospital of Guangzhou University of Chinese Medicine Guangzhou China

**Keywords:** dampness ZHENG, immune repertoire, psoriasis, rheumatoid arthritis, traditional Chinese medicine, ulcerative colitis

## Abstract

Autoimmune diseases, including psoriasis (Ps), rheumatoid arthritis (RA) and ulcerative colitis (UC), pose significant health burdens worldwide. A more refined classification of these diseases is essential for enabling targeted therapeutic strategies. Traditional Chinese Medicine (TCM) is gaining increasing recognition globally, offering potential insights into disease heterogeneity. In this study, we performed comprehensive T cell receptor (TCR) and B cell receptor (BCR) repertoire sequencing in 59 participants, including patients with Ps, RA, UC and healthy controls. Patients were further stratified into Dampness and non‐Dampness groups based on standardized TCM diagnostic criteria. Repertoire composition, clonotype richness, diversity metrics, V gene usage and shared CDR3 patterns were analysed. Machine learning approaches (LASSO, GLM and OPLS‐DA) were applied to identify diagnostic biomarkers, followed by validation in an independent cohort. Compared with healthy controls, Ps, RA and UC patients exhibited reduced clonotype richness and diminished TCR/BCR diversity, indicating adaptive immune contraction. Dampness ZHENG‐specific alterations were observed, including selective IgK and IgL clonotype richness reduction in Ps with dampness, increased TRA/TRB diversity in RA with dampness, and elevated TRG clonotype counts/read proportions in UC with dampness. Stratification by Dampness ZHENG revealed distinct immune repertoire architectures characterized by increased D50 index, reduced proportions of large clones and preferential V gene usage, including *TRAV20*, *TRAV38‐2DV8* and *TRAV8‐3*. Unsupervised dimensionality reduction demonstrated significant separation between Dampness and non‐Dampness groups across diseases. A three‐gene V‐segment model achieved an AUC of 0.804 and 74.7% accuracy in an independent validation cohort, supporting its potential utility as an objective biomarker for Dampness ZHENG. Our findings suggest that TCM‐based phenotyping may offer a meaningful approach for subgrouping autoimmune diseases, thereby providing a foundation for more syndrome differentiation‐based treatment strategies.

## Graphical abstract

Schematic diagram for the experimental design for adaptome immune repertoire analysing Dampness ZHENG across Psoriasis, Rheumatoid Arthritis and Ulcerative Colitis.

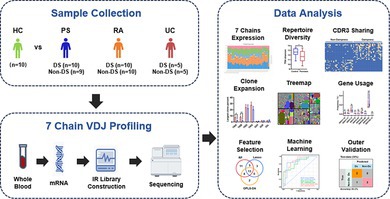

**Abbreviations**
AUC: area under curve
BCRs: B cell receptors
CDR3: complementarity‐determining region 3
DS: Dampness
IBDs: inflammatory bowel diseases
LASSO: LASSO regression
NGS: next‐generation sequencing
Non‐DS: non‐Dampness
OOB: the out‐of‐bag
OPLS‐DA: latent structures discriminant analysis
PBMC: peripheral blood mononuclear cell
Ps: psoriasis
RA: rheumatoid arthritis
RF: Random Forest
ROC: receiver operating characteristic curve
RT: reverse transcription
TCM: traditional Chinese medicine
TCRs: T cell receptors
Th17: T helper 17 cells
TRA: TCR‐alpha
TRB: TCR‐beta
TRD: TCR‐delta
Tregs: regulatory T cells
TRG: TCR‐gamma
UC: ulcerative colitis
uCDR3: clonotypes represent unique
UMI: unique molecular identifiers

## 1 Introduction

Autoimmune diseases comprise a heterogeneous group of disorders characterized by the breakdown of self‐tolerance, leading to activation of autoreactive T and B lymphocytes and the production of pathogenic autoantibodies [1, 2, 3]. Recent epidemiological studies estimate a global prevalence of 7.6%–9.4%, suggesting that nearly one in 10 individuals is affected by an autoimmune condition. These disorders may involve systemic or organ‐specific manifestations and display diverse clinical and immunological phenotypes, as exemplified by rheumatoid arthritis (RA), type 1 diabetes, systemic lupus erythematosus, psoriasis (Ps) and ulcerative colitis (UC). Their aetiology is multifactorial, encompassing genetic susceptibility, epigenetic modifications, environmental triggers and dysregulated innate and adaptive immune responses [4, 5]. Consequently, comprehensive profiling of the adaptive immune system is essential to elucidate disease mechanisms and identify diagnostic and therapeutic targets.

Psoriasis (Ps) is a common chronic inflammatory skin condition, impacting over 60 million individuals worldwide [6]. Psoriasis is Clinically characterized by salmon‐pink plaques with silvery scales. Its key histopathological features include aberrant keratinocyte hyperproliferation (hyperplasia) in the epidermis and a pervasive inflammatory cell infiltrate in the dermis [7]. A central immunopathogenic mechanism involves an imbalance between hyperactive T helper 17 (Th17) cells and functionally impaired regulatory T cells (Tregs) [8]. In addition, reduced frequencies of total B cells—particularly IL‐10–producing regulatory B cells (Bregs)—have been reported in moderate‐to‐severe disease [9].

Rheumatoid arthritis (RA) is another prevalent autoimmune disorder with a global prevalence of 0.5%–2.0% [10]. The disease is characterized by clinical manifestations such as joint destruction and functional disability, which consequently lead to a significant decline in quality of life [11]. Accumulating evidence implicates T and B cell dysfunction in RA pathogenesis. Notably, aberrantly activated autoreactive CD4^+^ T cells accumulate in circulation and target tissues, where they secrete pro‐inflammatory cytokines and promote autoreactive B cell activation, thereby amplifying synovial inflammation and tissue damage [12, 13].

Ulcerative colitis, a major subtype of inflammatory bowel disease (IBD), affects approximately 1.3% of the United States population [14]. Multiple studies link the pathogenesis of UC to aberrant innate and adaptive immune responses, particularly hyper‐reactions against microbiome and dietary antigens [15, 16]. The resulting mucosal inflammation is associated with increased T cell activation and maturation [16, 17]. Patients exhibit enhanced activation and differentiation of pro‐inflammatory T cell subsets (e.g., Th2, Th17, Th9), reduced regulatory populations (Tregs and Bregs), and increased cytotoxic CD8^+^ T cell–mediated tissue injury [18, 19, 20]. Supporting the role of antigen‐driven immunity, clonal expansion of TCR‐β has been observed in intestinal tissues of paediatric UC patients and correlates with disease severity [21].

Traditional Chinese Medicine (TCM) offers a complementary diagnostic framework based on the principle of ZHENG (pattern) differentiation, which integrates inspection, auscultation and olfaction, inquiry and pulse examination to achieve holistic classification. A frequently encountered pattern across diverse chronic conditions is Dampness ZHENG. Dampness ZHENG refers broadly to a class of syndromes resulting from dampness pathogen invading the skin, muscles, intestinal tract and joints, thereby obstructing qi and blood, or accumulating in the internal organs and impairing the functional activities of qi. As a longstanding tenet in Chinese medicine holds, ‘all diseases are attributable to dampness’. The dampness pathogen may attack various sites of the body, producing a spectrum of manifestations that progress from the exterior inward: affection of the skin gives rise to erythematous, scaly papules accompanied by itching or infiltration, as seen in psoriasis; involvement of the joints leads to stiffness, swelling and pain, as seen in rheumatoid arthritis; and retention in the viscera and bowels results in diarrhoea and passage of mucus, as seen in ulcerative colitis [22, 23, 24]. Although in real‐world TCM clinical practice these diseases often present as complex patterns centered on dampness (such as damp‐heat, intermingled dampness and blood stasis, or dampness arthralgia) [25, 26], we do not limit ourselves to any specific dampness‐centered complex pattern in this context. Rather, based on our Dampness Pattern Scale (Table 2), we place greater emphasis on the broader characteristics of the dampness pattern. Although clinicians can identify Dampness ZHENG based on its clinical manifestations (such as sleepiness, fatigue, sensation of head and so on), the intrinsic immunological and molecular basis underlying Dampness ZHENG remains largely undefined.

T and B lymphocytes mediate adaptive immunity through antigen‐specific recognition by T cell receptors (TCRs) and B cell receptors (BCRs) [27]. The hypervariable complementarity‐determining region 3 (CDR3), generated through V (variable)/D (diversity)/J (joining) gene recombination and junctional diversification, confers antigen specificity and serves as a molecular fingerprint of clonal expansion [27, 28]. Thus, the CDR3 regions of TCRs and BCRs are fundamental to understanding adaptive immunity in both physiological and pathological contexts [28], including autoimmune diseases. Immune repertoire sequencing has therefore emerged as a powerful approach to dissect adaptive immune dynamics in health and disease. While repertoire alterations have been extensively investigated in lupus nephritis [29], systemic lupus erythematosus [30, 31], and Primary Sjögren's Syndrome [32]. However, the comprehensive characterization of immune repertoire in psoriasis, RA and UC‐ particularly in relation to TCM‐defined patterns‐remains limited.

In the present study, we systematically profiled TCR and BCR repertoires in patients with Ps, RA and UC. We further compared immune repertoire features between Dampness and non‐Dampness ZHENG across these diseases to delineate pattern‐specific adaptive immune signatures. Through integrative analysis, we identified distinct clonotype distributions and receptor features that may serve as potential biomarkers for Dampness ZHENG. Overall, this study employs immune repertoire profiling to elucidate the pathogenesis of Ps, RA and UC. Simultaneously, it leverages these immune signatures to refine disease classification at the level of TCM syndromes. The insights gained from this integrated approach are poised to advance the framework for precision medicine in these diseases.

## 2 Material and Methods

### 2.1 Research Design and Methods

This current study was approved by the Ethical Committee of Guangdong Provincial Hospital of Chinese Medicine (No. BF2020‐075‐01), and all associated procedures were carried out in accordance with approved guidelines. A training cohort of 19 Ps patients, 20 RA patients, 10 UC patients (*n* = 49; Dampness = 25, non‐Dampness = 24) and 10 healthy subjects were included in the present study (Tables 1, 2, 3, 4, 5). Additionally, an external validation cohort of 30 Ps patients, 42 RA patients, 11 UC patients (*n* = 83; Dampness = 57, non‐Dampness = 26) was also recruited from Guangdong Provincial Hospital of Chinese Medicine (Table 6). One specialist in TCM from each of the departments of dermatology, rheumatology and gastroenterology, all possessing proficiency in Western diagnostic criteria, was involved in the diagnosis of Ps, RA and UC, respectively. The Ps patients were diagnosed with psoriasis vulgaris according to the hallmark histopathologic features and Psoriasis Area and Severity Index (PASI) [33, 34]. The RA patients fulfilled the 2010 ACR/European League Against Rheumatism classification criteria for RA [35]. The UC patients were diagnosed following the Consensus on diagnosis and treatment of inflammatory bowel disease [36]. All enrolled patients were in the stable phase of their disease. Furthermore, none of the enrolled patients had a recent infection. To minimize the potential influence of medications on immune profiling, all participants underwent a predefined washout period before sample collection.

**TABLE 1:** Summary of demographic characteristics in healthy individuals, psoriatic patients, RA patients and ulcerative colitis patients.

	Healthy individuals	Psoriatic patients	RA patients	UC patients
Number	10	19	20	10
Men	6	12	7	9
Women	4	7	13	1
Age, median (range)	37 (24–58)	46.63 (25–65)	45.35 (31–69)	41.2 (25–56)

Abbreviations: RA, rheumatoid arthritis; UC, ulcerative colitis.

**TABLE 2:** The diagnostic standards of Dampness TCM.

Clinical symptoms	Degree				
1. Obesity	Absence	Mild	Moderate	Severe	Very severe
2. Heavy body and Drowsiness	Absence	Mild	Moderate	Severe	Very severe
3. Hiding fever	Absence	Mild	Moderate	Severe	Very severe
4. Sticky sweating	Absence	Mild	Moderate	Severe	Very severe
5. Fatigue	Absence	Mild	Moderate	Severe	Very severe
6. Sleepiness	Absence	Mild	Moderate	Severe	Very severe
7. Heavy sensation of head	Absence	Mild	Moderate	Severe	Very severe
8. Greasy face or greasy hair	Absence	Mild	Moderate	Severe	Very severe
9. Dirty face	Absence	Mild	Moderate	Severe	Very severe
10. Excessive eye excrement	Absence	Mild	Moderate	Severe	Very severe
11. Sticky and greasy in mouth	Absence	Mild	Moderate	Severe	Very severe
12. Bad breath	Absence	Mild	Moderate	Severe	Very severe
13. Thirst	Absence	Mild	Moderate	Severe	Very severe
14. Phlegm	Absence	Mild	Moderate	Severe	Very severe
15. Abdominal fullness	Absence	Mild	Moderate	Severe	Very severe
16. Poor appetite	Absence	Mild	Moderate	Severe	Very severe
17. Nausea or vomit	Absence	Mild	Moderate	Severe	Very severe
18. Heavy drowsiness of waist	Absence	Mild	Moderate	Severe	Very severe
19. Joint sour pains	Absence	Mild	Moderate	Severe	Very severe
20. Heavy joints	Absence	Mild	Moderate	Severe	Very severe
21. Irregular stools	Absence	Mild	Moderate	Severe	Very severe
22. Increased defecation	Absence	Mild	Moderate	Severe	Very severe
23. Sticky stools	Absence	Mild	Moderate	Severe	Very severe
24. Increase leukorrhea/damp scrotum	Absence	Mild	Moderate	Severe	Very severe
25. Skin ulcer and sore	Absence	Mild	Moderate	Severe	Very severe
26. Thick coated tongue	Absence	Mild	Moderate	Severe	Very severe
27. Greasy coated tongue	Absence	Mild	Moderate	Severe	Very severe
28. Moist tongue with saliva	Absence	Mild	Moderate	Severe	Very severe
29. Line on the tongue	Absence	Mild	Moderate	Severe	Very severe
30. Plump tongue	Absence	Mild	Moderate	Severe	Very severe

*Note:* Evaluation standards: (1) Absence: 1 score; (2) Mild: 2 score; (3) Moderate: 3 score; (4) Severe: 4 score; (5) Very severe: 5 score. If the score reaches more than 15 points, the patients can be judged with Dampness ZHENG.

**TABLE 3:** Demographic and clinical characteristics of the psoriatic patients.

	Age	Sex	PASI	BSA	Dampness scores
Patient 1	55	M	4.5	9.4	22
Patient 2	58	M	9.3	10.2	18
Patient 3	29	F	6.5	8.2	30
Patient 4	41	F	4.2	4.5	26
Patient 5	59	F	4.6	7.5	19
Patient 6	43	M	6.8	8.1	23
Patient 7	65	M	3.7	6.51	21
Patient 8	61	M	7.5	9.7	29
Patient 9	47	M	3.6	7.05	34
Patient 10	53	F	5.3	3.87	15
Patient 11	26	M	3.1	3.3	11
Patient 12	34	M	2.1	2.1	8
Patient 13	41	F	2.5	3.9	6
Patient 14	38	M	6.5	15.4	4
Patient 15	61	F	4.4	8.6	5
Patient 16	51	M	4.4	6.7	10
Patient 17	48	M	7.6	17	9
Patient 18	25	M	2.8	1.7	8
Patient 19	51	F	0.3	0.12	7

*Note:* BSA, body surface area; PASI, psoriasis area and severity index.

**TABLE 4:** Demographic and clinical characteristics of the rheumatoid arthritis patients.

	Age	Sex	RF	Esr	hs‐CRP	DAS28	Dampness scores
Patient 1	46	F	304	13	0	2.63	20
Patient 2	59	M	14	54	5.19	3.14	15
Patient 3	60	F	31	18	1.78	2.98	25
Patient 4	50	F	99	76	6.68	2.86	24
Patient 5	67	M	177	76	6.68	3.13	15
Patient 6	60	F	11	15	0.66	2.73	15
Patient 7	41	F	174	23	3.78	3.12	17
Patient 8	40	F	40	24	1.09	2.67	21
Patient 9	67	F	49	78	1.73	2.7	18
Patient 10	67	F	46	92	3.74	3.16	17
Patient 11	31	F	217	≥ 120	21	3.17	12
Patient 12	69	M	38	40	5.39	3.16	6
Patient 13	63	M	13	82	6.89	2.71	8
Patient 14	53	M	676	23	2.18	2.76	10
Patient 15	47	F	40	3	1.02	2.78	7
Patient 16	61	M	7	34	13.12	3.17	9
Patient 17	66	F	11	68	14.39	3.18	10
Patient 18	44	F	20	20	0.83	2.61	13
Patient 19	50	F	165	18	0.09	3.01	8
Patient 20	60	M	109	38	3.12	2.63	11

Abbreviations: DAS, disease activity score; Esr, erythrocyte sedimentation rate; hs‐CRP, hypersensitive‐c‐reactive‐protein; RF, rheumatoid factor.

**TABLE 5:** Demographic and clinical characteristics of the ulcerative colitis patients.

	Age	Sex	Mayo scores	Dampness scores
Patient 1	44	M	8	31
Patient 2	25	M	5	18
Patient 3	56	M	6	26
Patient 4	38	M	3	35
Patient 5	44	M	3	19
Patient 6	44	M	4	8
Patient 7	41	M	3	14
Patient 8	26	M	6	11
Patient 9	34	F	3	3
Patient 10	24	M	3	6

**TABLE 6:** A separate group of newly recruited patients for an external validation cohort.

Patient	Disease	Age	Sex	PASI	BSA	Dampness scores
1	Pso	33	M	4.4	8.1	21
2	Pso	43	M	4.9	7	15
3	Pso	52	M	5.8	12.9	17
4	Pso	23	M	6.2	4.75	22
5	Pso	45	M	5.1	4.15	26
6	Pso	30	M	7.4	20.4	20
7	Pso	26	M	5.7	5.38	15
8	Pso	22	M	7.3	13.4	19
9	Pso	29	M	6	3.7	24
10	Pso	30	M	3.9	6.01	22
11	Pso	24	F	4.1	3.7	23
12	Pso	25	F	4	5	29
13	Pso	37	M	3.7	0.78	16
14	Pso	52	F	8.1	5.9	18
15	Pso	30	F	6	10.6	30
16	Pso	61	M	5.6	5	22
17	Pso	33	M	3.6	3.85	25
18	Pso	38	M	4.2	9.22	20
19	Pso	42	M	3	3.84	18
20	Pso	37	M	5.1	3.05	19
21	Pso	19	M	3.9	4.5	11
22	Pso	22	F	7.8	15.7	7
23	Pso	46	F	3.7	3.8	6
24	Pso	53	M	7.1	14.5	9
25	Pso	39	M	6.9	10.5	10
26	Pso	28	F	2	0.9	12
27	Pso	25	F	0.8	1.96	3
28	Pso	48	M	3.6	4	8
29	Pso	31	M	3.6	4	10
30	Pso	61	F	3.1	6.5	4

Patient	Disease	Age	RF	Sex	Esr	CRP	DAS28	Dampness scores
1	RA	37	26	F	26	78.9	2.7	17
2	RA	55	12	F	12	11.7	2.7	25
3	RA	34	11	M	11	8	2.7	28
4	RA	63	18	F	18	23.4	3.0	21
5	RA	58	5	F	5	0.5	2.9	18
6	RA	35	3	F	3	0.5	3.0	16
7	RA	67	10	F	10	64.5	2.8	23
8	RA	29	8	F	8	1.2	3.2	26
9	RA	46	51	M	51	200	3.0	18
10	RA	62	169	F	169	35.3	3.0	22
11	RA	54	7	F	33	0	2.6	27
12	RA	70	50	F	38	2.22	3.2	24
13	RA	57	8	F	24	3.52	2.9	19
14	RA	51	98	F	43	0.66	3.0	17
15	RA	53	73	F	30	2.85	2.9	29
16	RA	59	20	F	9	0.01	2.7	21
17	RA	38	97	F	88	26.82	3.1	15
18	RA	27	19	F	28	5.16	3.1	22
19	RA	55	116	M	32	3.86	3.2	26
20	RA	51	70	F	47	0.64	2.9	18
21	RA	57	8	F	10	0.01	2.7	15
22	RA	54	29	F	42	9.67	3.1	19
23	RA	68	21	F	21	0	2.6	22
24	RA	42	98	F	73	3.67	3.0	25
25	RA	41	4	M	14	2.22	2.7	19
26	RA	63	8	F	11	0.34	3.0	15
27	RA	55	13	F	6	0	2.8	19
28	RA	65	112	M	9	0.01	2.7	21
29	RA	70	26	F	47	0.7	2.5	14
30	RA	46	147	F	39	0.95	3.0	6
31	RA	71	10	M	98	2.36	3.2	8
32	RA	40	6	M	4	1.22	3.3	10
33	RA	39	7	F	37	1.17	3.0	11
34	RA	44	200	F	51	7.72	4.8	13
35	RA	68	11	F	120	36.31	6.5	7
36	RA	50	201	M	14	1.29	3.6	5
37	RA	43	16	F	24	3.85	4.0	8
38	RA	37	16	F	59	15.41	5.5	9
39	RA	46	8	F	10	0.17	2.5	6
40	RA	51	72	F	95	0.35	1.2	12
41	RA	52	120	F	19	4.1	4.4	4
42	RA	46	26	F	6	0.1	2.4	9

Patient	Disease	Age	Sex	Mayo scores	Dampness scores
1	UC	28	M	4	23
2	UC	25	M	6	36
3	UC	28	M	3	28
4	UC	27	M	4	17
5	UC	31	M	4	25
6	UC	26	F	2	19
7	UC	25	F	5	15
8	UC	22	M	6	29
9	UC	37	M	3	22
10	UC	64	M	6	13
11	UC	42	F	8	10

Abbreviations: Pso, psoriasis; RA, rheumatoid arthritis; UC, ulcerative colitis.

Subsequently, these doctors performed dampness pattern differentiation on the aforementioned patients with Ps, RA and UC according to the Diagnostic Standards of Dampness in TCM (Table 2). If the score reaches more than 15 points, the patients can be judged with Dampness ZHENG. The main clinical and epidemiological characteristics of the training cohort and the external validation cohort of Ps, RA and UC patients are summarized in Tables 3, 4, 5, 6. All participants were informed about the study and signed a consent form before being recruited into the study. Additionally, these doctors were kept blinded to the immune repertoire findings.

### 2.2 Sample Collection and RNA Extraction

All the blood samples were collected from patients with informed consent. Two millilitres of fresh blood samples were drawn into PAXgene Blood RNA Tube (Qiagen, Germany, Cat. No. 762165) via venipuncture and the samples were placed at room temperature for 4 h before being transferred to −80°C. Total RNA was then extracted from these samples using PAXgene Blood RNA Kit (Qiagen, Germany, Cat. No. 762331), following manufacturer's instructions.

### 2.3 TCR and BCR Immune Repertoire Sequencing

Immune repertoire sequencing was performed following previously published protocols [37]. Extracted total RNA samples were further processed for construction of TCR/BCR libraries using commercially available iR‐RepSeq plus 7‐Chain Cassettes (iRepertoire Inc., USA, Cat. No. iR + 7chain‐HLRI‐C) covering all seven human TCR and BCR chains, allowing incorporation of unique molecular identifiers (UMI) during the reverse transcription (RT) step. Each sample was processed in one cassette, of which was preloaded with all library preparation, including Qiagen OneStep RT PCR mix (Qiagen) and SPRIselect bead selection (Beckman Coulter) to remove the unused primers and first‐strand synthesis. Library amplification is performed with a pair of primers that are specific for communal sites engineered onto the 5′ end of the C‐ and V‐ primers used in the first and second strand synthesis. The final constructed library includes illumina dual index sequencing adapters, a 10‐nucleotide unique molecular identifier region, and an 8‐nucleotide internal barcode associated with the C‐gene primer.

Libraries that underwent amplification were multiplexed and pooled for sequencing on the Illumina NovaSeq platform. The use of 300 base‐pair paired‐end sequencing allowed complete coverage of the full variable region, including CDR1, CDR2 and CDR3. The raw sequences obtained were collapsed by 10‐bp UMI tags into the consensus FASTA format using MiGEC version 1.2.9 (https://migec.readthedocs.io/en/latest/) and were analysed using the iRmap program [38]. The QC information (such as RNA quality metrics, sequencing depth/UMI counts per sample) of immune repertoire sequencing is presented in Excels S1 and S2. In the preprocessing stage, we removed all non‐productive mapping entries, so the productive ratio in our analysis is 100%. We performed UMI‐based collapse, so sequencing depth does not affect diversity or richness.

Briefly, sequence reads were de‐multiplexed according to both Illumina dual indices incorporated during the amplification process and barcode sequences at the 5′ end of reads from the constant region. For immune repertoire genes sequencing, paired‐end FASTA were demultiplexed by 6 bp barcode using MiGEC version 1.2.9 and then stitched into a single read using pandaseq version 2.11. The merged reads were mapped using a Smith‐Waterman algorithm to germline V, D, J and C reference sequences using an IMGT reference library. To define the CDR3 region, the position of CDR3 boundaries of reference sequences from the IMGT database was migrated onto reads through mapping results, and the resulting CDR3 regions were extracted and translated into amino acids. The dataset was condensed by the combination of UMIs and CDR3 regions to remove incorrect CDR3s introduced by sequencing and amplification. Reads with the same combination of CDR3 and UMI were condensed into one. The raw sequence data reported in this paper have been deposited in the Genome Sequence Archive (Genomics, Proteomics & Bioinformatics 2021) in National Genomics Data Center (Nucleic Acids Res 2021), China National Center for Bioinformation/Beijing Institute of Genomics, Chinese Academy of Sciences (GSA: HRA013682) that are publicly accessible at https://ngdc.cncb.ac.cn/gsa.

### 2.4 Machine Learning Model Development and Evaluation

Patients were enrolled sequentially into two independent cohorts. The training cohort (*n* = 49; Dampness = 25, non‐Dampness = 24) was recruited during the initial study period and used for feature selection and model construction (Tables 3, 4, 5). After the training cohort was completed, a separate group of newly recruited patients was prospectively collected to form an external validation cohort (*n* = 83; Dampness = 57, non‐Dampness = 26) (Table 6). No individual was included in both cohorts, ensuring temporal separation and preventing data reuse. No randomized or stratified splitting was applied; instead, the validation cohort served exclusively for independent assessment of model generalizability.

We first employed LASSO regression, generalized linear models (GLM) and orthogonal partial least squares discriminant analysis (OPLS‐DA) to identify characteristic V genes that distinguish Dampness from Non‐Dampness individuals in the training cohort.

Because the cohort contained multiple disease categories, disease classification was included as a covariate to reduce potential confounding. Disease labels were one‐hot encoded and incorporated into the regression models. For LASSO feature selection, LASSO logistic regression (family = binomial, alpha = 1) was performed using the glmnet package with 10‐fold cross‐validation. To ensure disease effects were adjusted rather than selected, the disease covariates (including the intercept) were forced into the model by setting their penalty factors to 0, while V‐gene variables were penalized (penalty factor = 1). Features with non‐zero coefficients at the optimal *λ* (λ.min) were retained.

In parallel, OPLS‐DA was conducted using the ropls package with Pareto scaling and 1000 permutation tests to assess overfitting. Variables with VIP scores greater than 1.5 were considered discriminative.

Additionally, each V‐gene feature was tested using a GLM adjusting for disease category (feature ~ group + disease). For features with values in the 0–1 range, a logit transformation was applied prior to modelling. Multiple testing correction was performed using the Benjamini–Hochberg procedure, and features with FDR < 0.1 were considered significant.

Robust candidate biomarkers were defined as the intersection of all three selection strategies (LASSO non‐zero, OPLS‐DA VIP > 1.5, GLM FDR < 0.1), yielding four V genes (as summarized in the Venn diagram). These candidates were carried forward for downstream evaluation. For each of these genes, receiver operating characteristic (ROC) curves were plotted using the multipleROC package, and the corresponding areas under the curve (AUCs) were calculated. The three genes with the highest AUC values were selected for diagnostic model construction.

To evaluate classification performance, multiple machine learning models were trained to distinguish Dampness from Non‐Dampness using the selected V gene frequencies as predictors. Preprocessing was performed using a recipe that specified the Dampness group as the outcome and the selected V genes as predictors. Five algorithms were assessed through the tidymodels framework: logistic regression, decision tree, random forest, K‐nearest neighbours (KNN) and XGBoost, each implemented with its corresponding classification engine. For each algorithm, a unified workflow was created by combining the preprocessing recipe with the model specification.

Model performance was estimated using 1000 bootstrap resampling iterations, with out‐of‐bag predictions retained for aggregation. AUC, accuracy and Brier score were calculated for comparative ranking. Logistic regression achieved the most favourable combination of high AUC and accuracy along with the lowest Brier score and was therefore selected as the final classifier.

Finally, the diagnostic performance of the selected V genes was evaluated in the external validation cohort. A logistic regression model was constructed using the glm function, and predicted probabilities were used to generate a confusion matrix and ROC curve through the pROC package. The resulting AUC value was annotated to assess discriminative ability in an unseen patient cohort.

### 2.5 Statistical Analysis

We employed the Mann–Whitney *U* test to analyze the expression percentage of reads number, the number or the diversity of uCDR3, the percentage of large clonal expansion, V and J gene usage frequencies. Additionally, we performed a quantitative analysis of unsupervised dimensionality reduction (UMAP) by comparing the intra‐group distances (distances between points within the same group) with the inter‐group Euclidean distances (distances between points from different groups). Moreover, the candidate biomarkers of Dampness ZHENG were defined as the intersection of all three selection strategies (LASSO non‐zero, OPLS‐DA VIP > 1.5, GLM FDR < 0.1). The data were presented as mean with standard deviation. All statistical analyses were performed using the statistical software SPSS for Windows, Version 26.0 and GraphPad Prism V 8.00.

## 3 Results

### 3.1 Cohort Characteristics

Fifty‐nine participants were enrolled, including 19 patients with psoriasis (Ps), 20 with rheumatoid arthritis (RA), 10 with ulcerative colitis (UC) and 10 age‐ and sex‐matched healthy controls. All subjects were recruited from Guangdong Provincial Hospital of Chinese Medicine. Diagnoses were established according to internationally recognized criteria (see Methods). Baseline demographic and clinical features are summarized in Tables 1 and 3, 4, 5. No significant differences in age or sex distribution were observed between patient groups and controls.

### 3.2 Convergent Contraction of Adaptive Immune Repertoires in Ps, RA and UC

**FIGURE 1:**
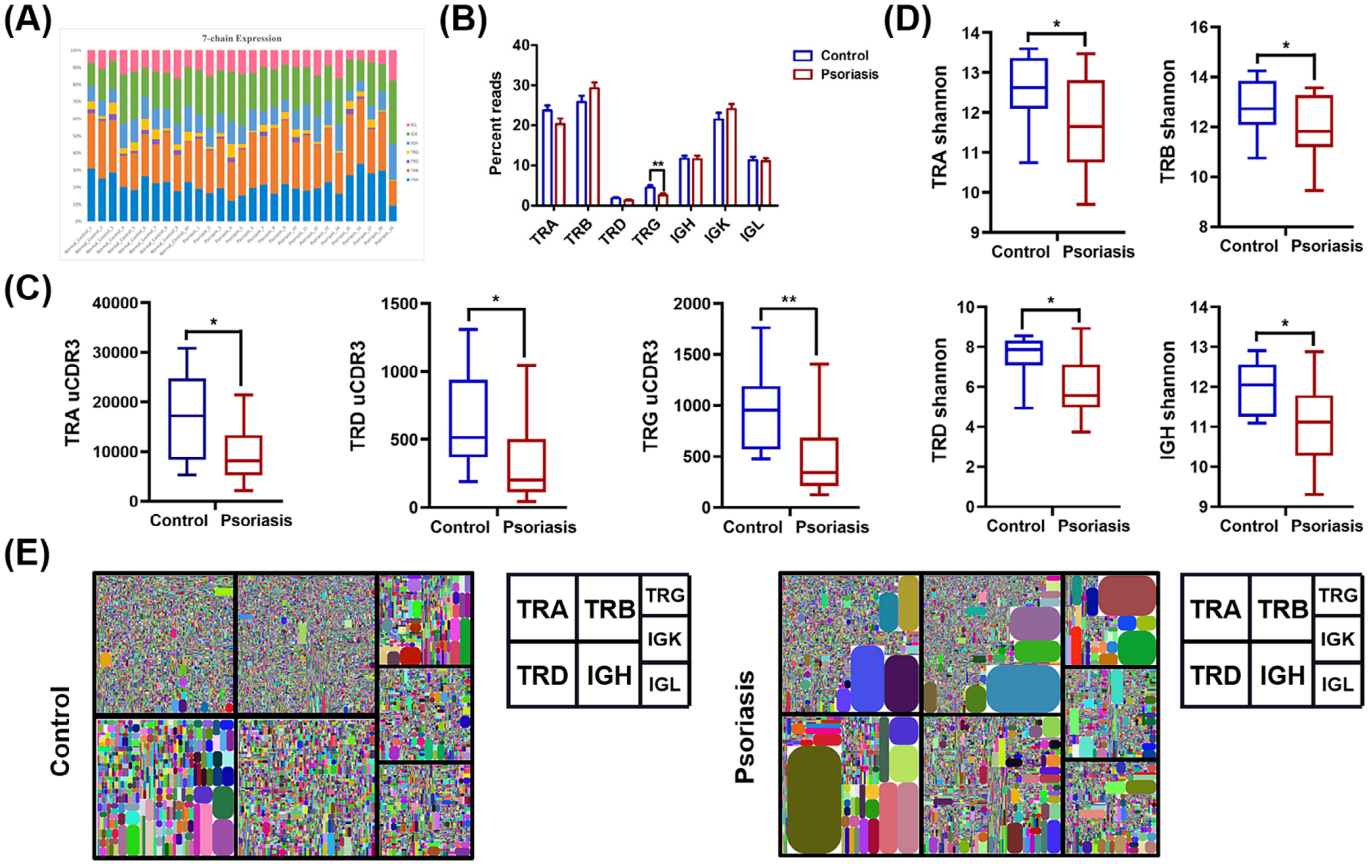
The distinct characterizations of TCR and BCR immune repertoire between Ps patients and healthy controls. Data were collected from 29 biological replicated experiments *n* = 19 for PS patients and *n* = 10 for healthy controls. (A) Each bar represents the composition ratio of seven chain immune repertoire in one sample (healthy controls, *n* = 10; PS patients, *n* = 19). (B) Expression percentage calculated by reads in each chain from control subjects and psoriasis patients. (C) The abundance of each chain for immune repertoire adaptome is measured by the number of uCDR3, and uCDR3 counts of TRA, TRD and TRG are presented for two groups as mean value ± SD in the boxplots. (D) The diversity was demonstrated with shannon‐index at the level of unique uCDR3 clones, as shown for TRA, TRB, TRD and IgH. (E) Seven‐chain repertoire tree maps for two representative subjects from each group. Each square represents a chain. All tree maps should be read from left to right and then from top to bottom in the following order: TRA, TRB, TRD, IGH, TRG, IGK and IGL. Each rounded rectangle colour block is a clone. The Mann–Whitney *U* test was used for B, C and D. Ps, psoriasis. *p < 0.05, **p < 0.01. Data represent the mean ± S.D.

**FIGURE 2:**
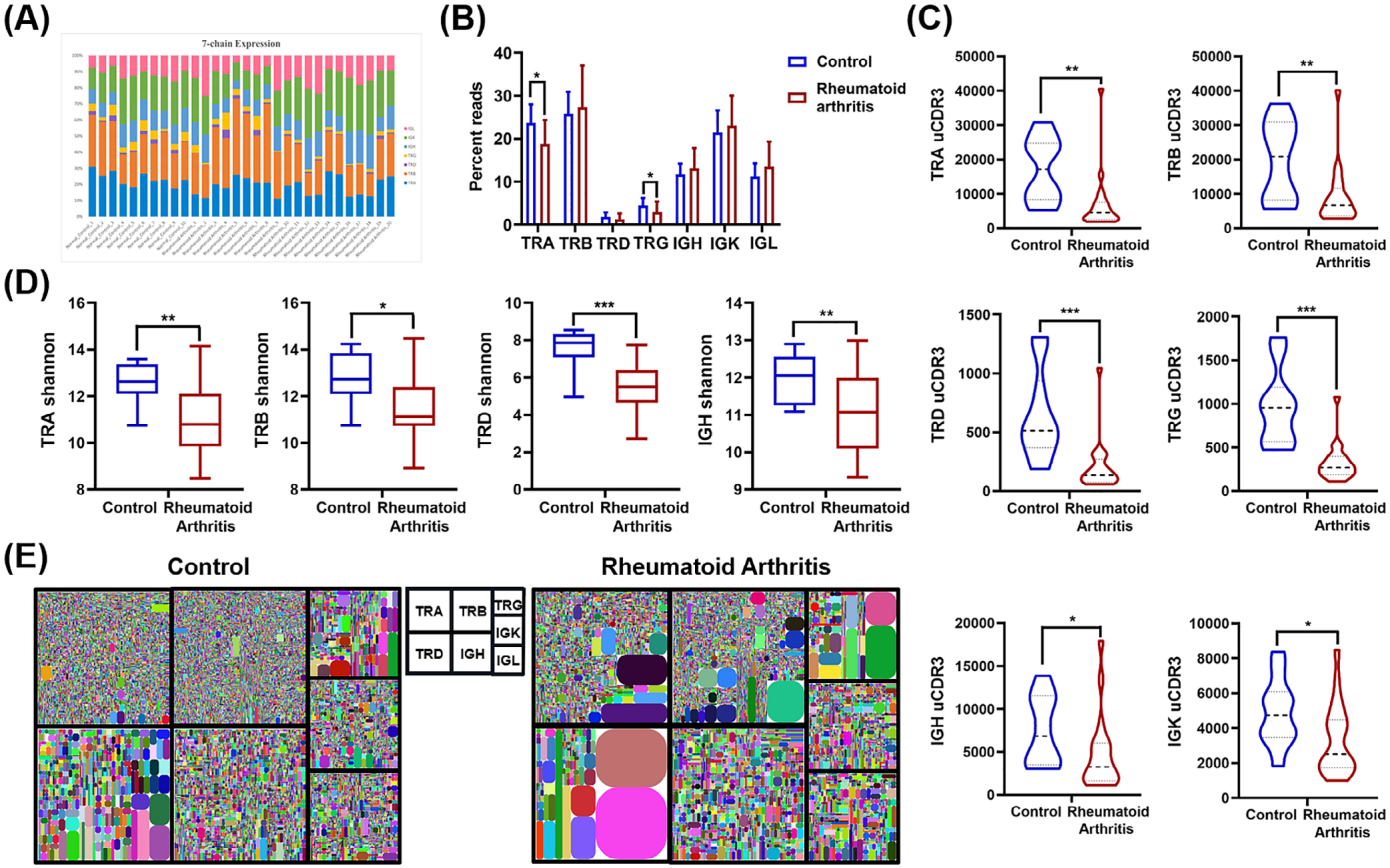
The differential features of TCR and BCR immune repertoire between RA patients and healthy control. Data were collected from 30 biological replicated experiments *n* = 20 for RA patients and *n* = 10 for healthy controls. (A) Each bar represents the composition ratio of seven chain immune repertoire in one sample (healthy controls, *n* = 10; RA patients, *n* = 20). (B) Expression percentage calculated by reads in each chain from control subjects and RA patients. (C) The abundance of each chain for immune repertoire adaptome is measured by the number of uCDR3 counts for TRA, TRB, TRD, TRG, IgH and IgK. (D) The diversity was demonstrated with shannon‐index as shown for TRA, TRB, TRD and IgH. (E) Seven‐chain repertoire tree maps for two representative subjects from each group. Each square represents a chain. All tree maps should be read from left to right and then from top to bottom in the following order: TRA, TRB, TRD, IGH, TRG, IGK and IGL. Each rounded rectangle colour block is a clone. RA, rheumatoid arthritis. Mann–Whitney *U* test was used for B, C and D. *p < 0.05, **p < 0.01, ***p < 0.001. Data represent the Mean ± S.D.

**FIGURE 3:**
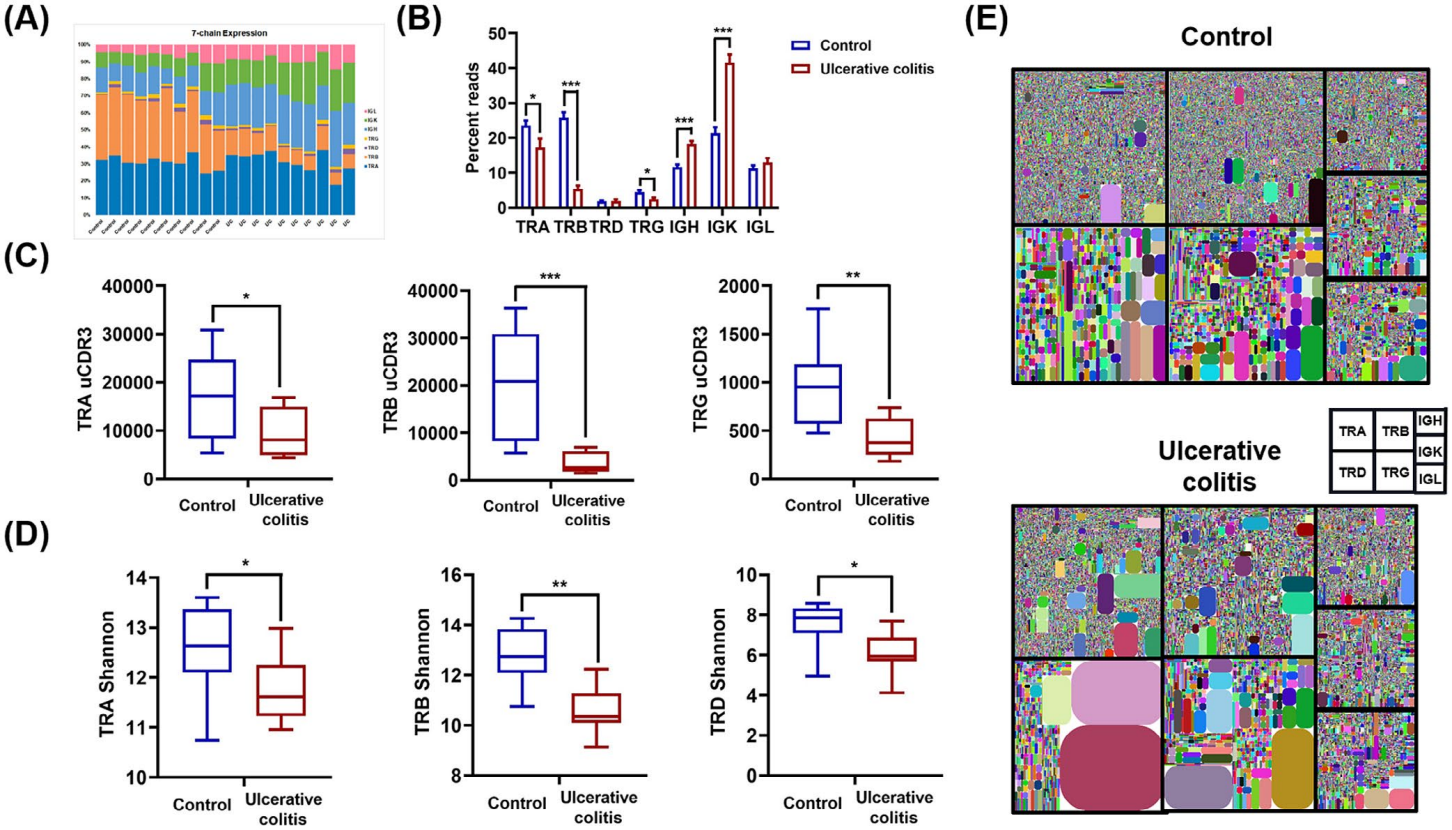
The differential pattern of TCR and BCR immune repertoire between UC patients and healthy control. Data were collected from 20 biological replicated experiments *n* = 10 for UC patients and *n* = 10 for healthy controls. (A) Each bar represents the composition ratio of seven chain immune repertoire in one sample (healthy controls, *n* = 10; UC patients, *n* = 10). (B) Expression percentage calculated by reads in each chain from control subjects and UC patients. (C) The abundance of each chain for immune repertoire adaptome is measured by the number of uCDR3 counts for TRA, TRB and TRG. (D) The diversity was demonstrated with shannon‐index as shown for TRA, TRB and TRD. (E) Seven‐chain repertoire tree maps for two representative subjects from each group. Each square represents a chain. All tree maps should be read from left to right and then from top to bottom in the following order: TRA, TRB, TRD, TRG, IGH, IGK and IGL. Each rounded rectangle colour block is a clone. UC, ulcerative colitis. Mann–Whitney *U* test was used for B, C and D. *p < 0.05, **p < 0.01, ***p < 0.001. Data represents the Mean ± S.D.

To define global adaptive immune alterations, we first analysed TCR and BCR chain composition across diseases. Compared with healthy controls, Ps patients exhibited a selective reduction in TRG chain representation (Figure 1A,B), suggesting perturbation of γδ T cell compartments. RA patients demonstrated broader T cell repertoire contraction, particularly affecting TRA and TRG chains (Figure 2A,B), consistent with chronic antigen‐driven T cell activation. In UC, the immune imbalance was more pronounced: TCR chains (TRA, TRB, TRG) were significantly reduced, whereas BCR chains (IgH and IgK) were expanded (Figure 3A,B), indicating a shift toward humoral predominance in intestinal inflammation.

We next quantified clonotype richness using unique CDR3 (uCDR3) counts. Across diseases, patients exhibited significant reductions in uCDR3 numbers relative to controls. Ps showed decreased TRA, TRD and TRG clonotypes; RA exhibited widespread reductions across TCR and BCR chains; and UC displayed reduced TRA, TRB and TRG clonotypes (Figures 1, 2, 3).

Numerous studies have reported the correlation between human diseases and diminished immune repertoire diversity [39, 40, 41]. To further explore the repertoire feature of Ps, RA and UC patients, the diversity of TCR and BCR repertoires was measured in all samples. Shannon entropy describes the evenness and richness of the repertoire [42], and here we used Shannon entropy to measure the diversity of the immune repertoires. All three diseases demonstrated significantly reduced diversity across multiple chains (TRA, TRB, TRD and IgH) (Figures 1, 2, 3), accompanied by visually evident clonal skewing in treemap analyses (Figures 1, 2, 3).

Collectively, these data reveal convergent contraction and skewing of adaptive immune repertoires across Ps, RA and UC, consistent with persistent antigenic stimulation and restricted clonal expansion.

### 3.3 Dampness ZHENG Is Associated With Disease‐Specific Immune Remodelling

TCM phenotyping can identify distinct subgroups within patients already categorized by Western medicine disease criteria, adding a complementary layer of classification. In the TCM ideological system, Dampness is one of the major and most important ZHENG (syndromes). To determine whether Dampness ZHENG (DS) corresponds to distinct immunological states, patients within each disease were stratified into DS and non‐DS groups based on standardized TCM diagnostic criteria (Table 2).

**FIGURE 4:**
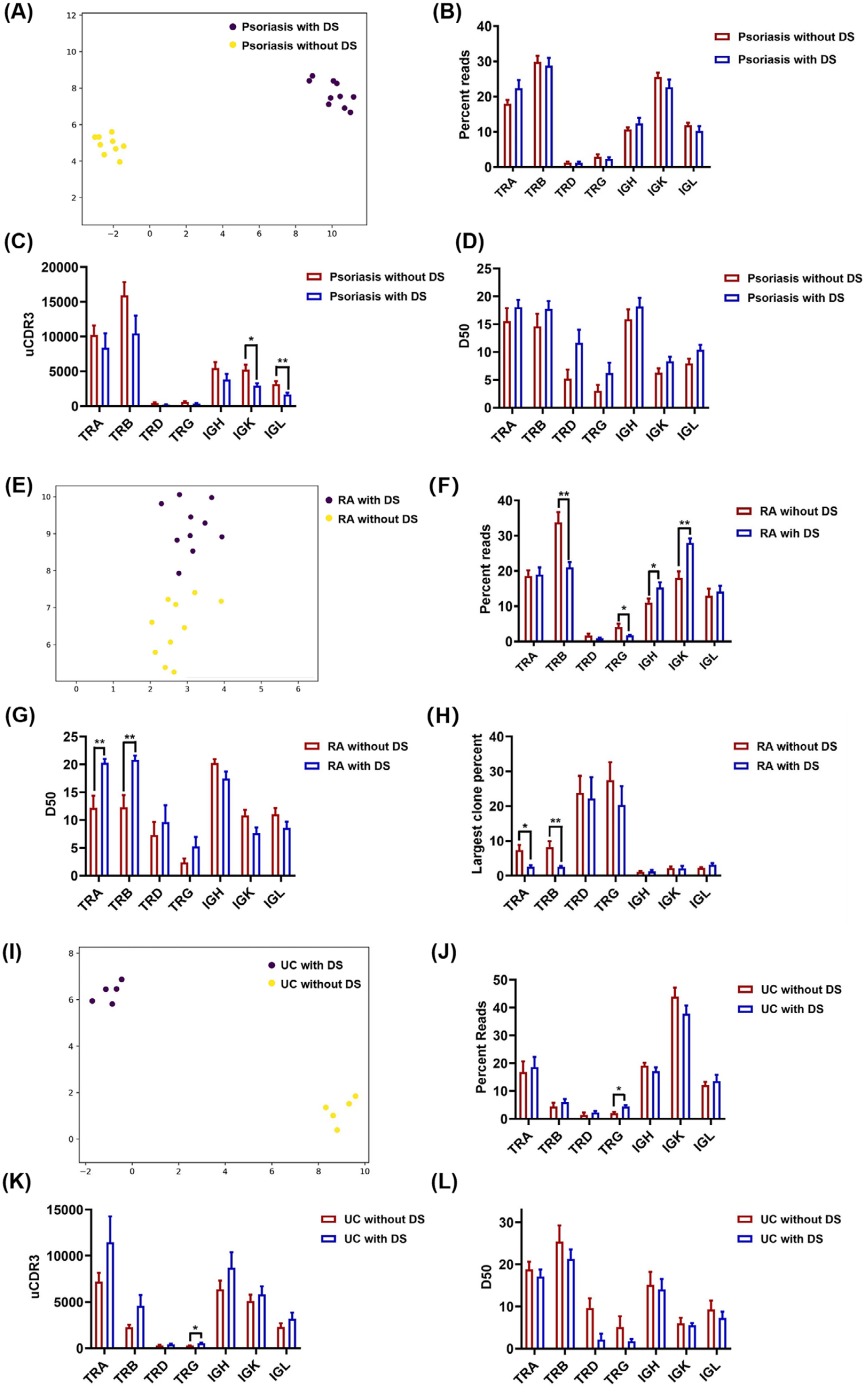
Immune repertoire feature of Dampness and Non‐Dampness Ps/RA/UC patients. (A‐D) UMAP, expression percentage calculated by reads in each chain, the number of uCDR3 counts and the diversity (D50) between Dampness Ps and non‐Dampness Ps patients, respectively. (E‐H) UMAP, expression percentage calculated by reads, the diversity (D50) and the largest clone percent between Dampness RA and non‐Dampness RA patients, respectively. (I‐L) UMAP, expression percentage calculated by reads in each chain, the number of uCDR3 counts and the diversity (D50) between Dampness UC and non‐Dampness UC patients, respectively. Mann–Whitney *U* test was used for B‐D, F‐H and G‐L. *p < 0.05, **p < 0.01. Data represents the Mean ± S.D. DS, Dampness. RA, rheumatoid arthritis; UC, ulcerative colitis.

In Ps, global repertoire composition did not differ significantly between DS and non‐DS groups; however, IgK and IgL clonotype richness was significantly reduced in DS patients (Figure 4B,C), suggesting selective B cell contraction. In RA, DS patients exhibited a coordinated shift characterized by reduced T cell (TRB, TRG) proportions and increased B cell (IGH, IGK) representation (Figure 4F). Notably, RA‐DS patients displayed fewer expanded large clones and increased TRA/TRB diversity (Figure 4G,H), indicating enhanced clonal evenness relative to non‐DS patients. In UC, DS was associated with increased TRG read proportions and elevated TRG clonotype counts (Figure 4j,k), implicating *γδ* T cell involvement in Dampness‐associated intestinal inflammation. In our results, the intra‐group point distances were markedly smaller than the inter‐group Euclidean distances among the Dampness and Non‐Dampness groups in Pso, RA and UC patients, suggesting pronounced differences in their immune repertoire features (Figure 4A,E,I) (Figure S1 and Excels S3–S5).

These findings indicate that Dampness ZHENG is not merely a clinical descriptor but corresponds to reproducible adaptive immune states.

### 3.4 Cross‐Disease Immune Signature of Dampness ZHENG

To identify syndrome‐specific signatures independent of Western disease classification, all Ps, RA and UC patients were regrouped by Dampness status.

**FIGURE 5:**
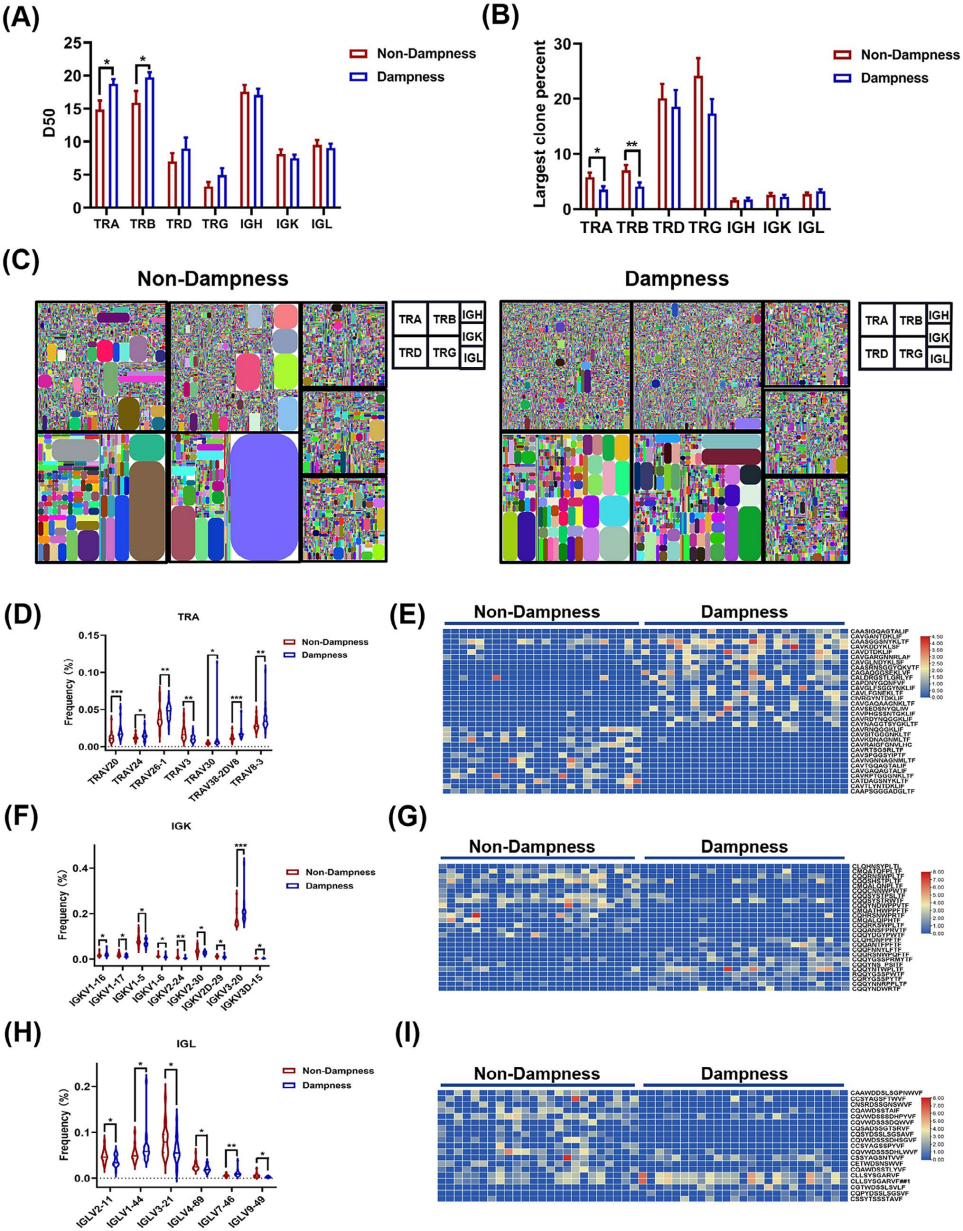
TCR/BCR adaptome immune repertoire pattern and signature of Dampness ZHENG. (A) The diversity was demonstrated with D50 between Dampness/Non‐Dampness patients. (B) The percentage of large clone between Dampness and Non‐Dampness patients. Large clones were defined as top 1% expression in each sample. (C) Seven‐chain repertoire tree maps for two representative subjects from each group. Each square represents a chain. All tree maps should be read from left to right and then from top to bottom in the following order: TRA, TRB, TRD, TRG, IGH, IGK and IGL. Preferred *TRAV* (D), *IGKV* (F) and *IGLV* (H) genes with *p* value < 0.05, between Dampness and Non‐Dampness are presented. TRA (E), IGK (G) and IGL (I) cluster analysis heat map for CDR3 sharing between Dampness and Non‐Dampness subjects. Each rounded rectangle colour block is a clone. D50 is calculated as the percentage of clones that make up the top 50% of reads in the ranked clone distribution. Mann–Whitney *U* test was used for A, B, D, F and H. *p < 0.05, **p < 0.01, ***p < 0.001. Data represents the Mean ± S.D.

Dampness patients exhibited a significantly elevated D50 index and reduced proportions of large clones (Figure 5A,B), indicating increased T cell repertoire evenness. Representative tree maps illustrate this redistribution of clonal architecture (Figure 5C). V gene usage analysis revealed distinct patterns associated with Dampness. Increased usage of *TRAV20*, *TRAV38‐2DV8* and *TRAV8‐3* was observed, whereas *TRAV3* and several IGKV/IGLV genes were reduced (Figure 5D,F,H). Analysis of shared CDR3 sequences further demonstrated differential enrichment of TRA, IGK and IGL clonotypes between groups (Figure 5E,G,I), suggesting antigen‐driven convergence or shared immune selection pressures underlying Dampness ZHENG.

Together, these data define a cross‐disease adaptive immune signature characteristic of Dampness ZHENG.

### 3.5 Predictive Modelling Identifies *TRAV20* , *TRAV38‐2DV8* and *TRAV8‐3* as Candidate Biomarkers for Dampness ZHENG

**FIGURE 6:**
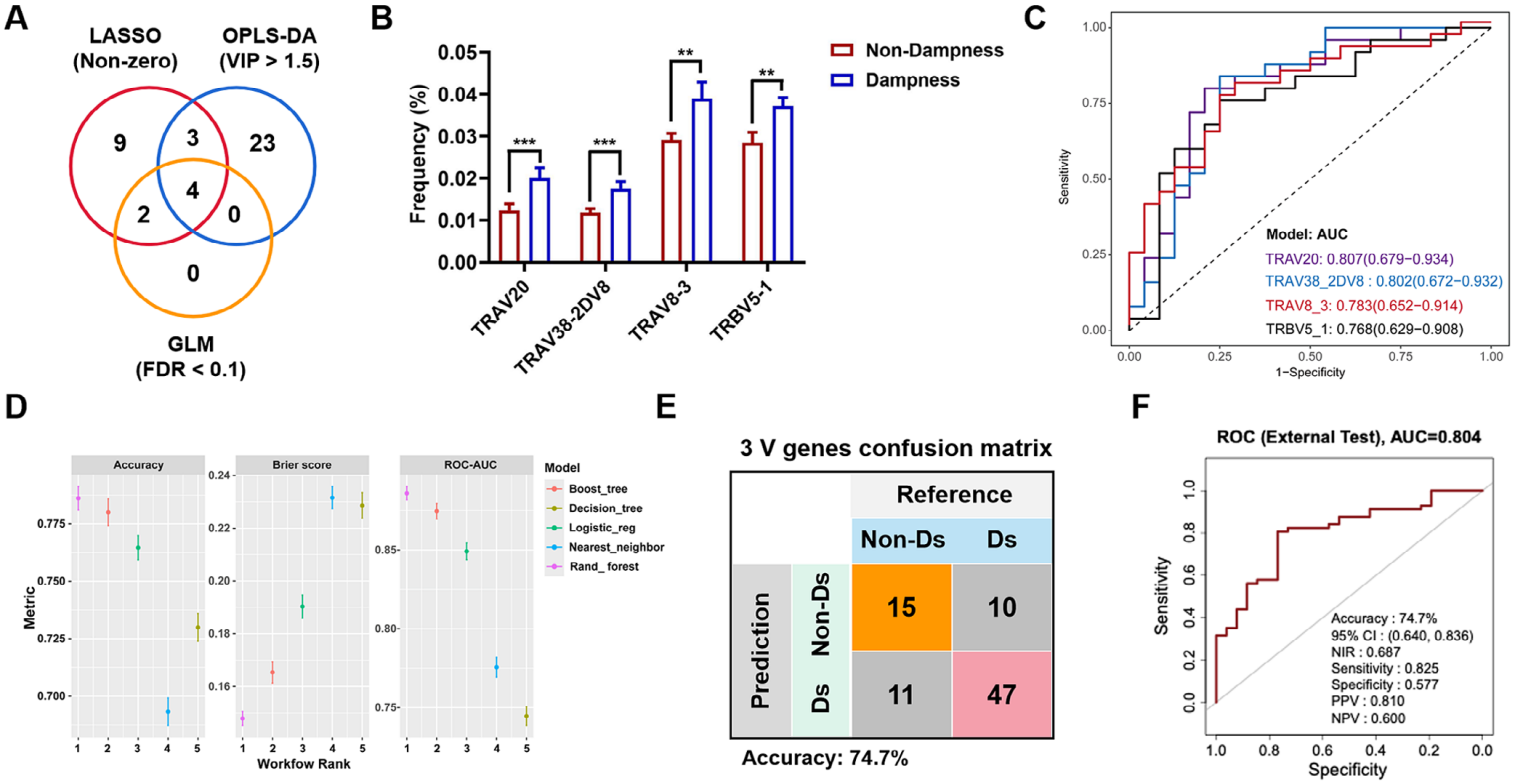
Identification and validation of key V genes as biomarkers for discriminating Dampness and Non‐Dampness. (A) The Venn diagram of V genes selected by LASSO, OPLS‐DA and GLM analysis. (B) The frequence of 4 selected V genes between Non‐Dampness and Dampness group. (C) ROC curve plot of 4 selected V genes with the AUC value. (D) Visualization of performance metrics across different machine learning models. (E) The confusion matrix of predictive model conducted by logistic regression using Top3 V genes. (F) The ROC curve of the predictive model. LASSO, LASSO regression; OPLS‐DA, orthogonal partial least squares discriminant analysis; GLM, generalized linear models; NIR, no information rate; PPV, positive predict value; NPV, Negative predict value. Mann–Whitney *U* test was used for B. **p < 0.01, ***p < 0.001. Data represents the Mean ± S.D.

To evaluate translational utility, we applied LASSO regression, GLM and OPLS‐DA within a training cohort (*n* = 49). Four V genes overlapped across all methods (Figure 6A), each significantly different between DS and non‐DS groups (Figure 6B).

ROC analysis identified *TRAV20*, *TRAV38‐2DV8* and *TRAV8‐3* as top‐performing markers (Figure 6C). Logistic regression achieved superior discrimination compared to alternative models, yielding the highest AUC and lowest Brier score (Figure 6D). In an independent validation cohort of 83 patients (57 Dampness, 26 Non‐Dampness) (Table 6), the three‐gene model achieved an accuracy of 74.7% and an AUC of 0.804 (95% CI = 0.640–0.836, sensitivity = 0.825, specificity = 0.577) (Figure 6E,F), supporting its robustness.

Immunohistochemical analysis of T and B cell infiltration in Ps and UC tissues revealed no significant differences between DS and non‐DS groups (Figure S2), indicating that repertoire alterations reflect clonal architecture rather than total lymphocyte abundance.

Overall, these findings establish *TRAV20*, *TRAV38‐2DV8* and *TRAV8‐3* as promising immune repertoire biomarkers for Dampness ZHENG and provide molecular support for TCM‐based phenotypic stratification.

## 4 Discussion

Autoimmune diseases arise from dysregulated self‐recognition and persistent activation of adaptive immune responses directed against host tissues [43]. Globally, nearly 4% of the population is affected by autoimmune conditions such as psoriasis, rheumatoid arthritis, type 1 diabetes and Crohn's disease. These diseases not only significantly impair quality of life but are also associated with a mortality rate of 15 per million. Immune repertoire sequencing has emerged as a powerful tool to interrogate these processes at high resolution, enabling the identification of disease‐associated clonotypes, V gene biases and signatures of antigen‐driven selection. Liu et al. identified characteristic disease‐associated TCR markers in lupus and rheumatoid arthritis [44], while Luo et al. reported a distinct hormonal immune profile accompanied by elevated IgE production in psoriasis patients [45]. Additionally, companion diagnostic biomarkers related to responsiveness to immunosuppressive drugs in lupus treatment have been identified, especially within BCR repertoires [46]. In this study, we performed comprehensive adaptome profiling across three autoimmune diseases, such as psoriasis, rheumatoid arthritis and ulcerative colitis.

Consistent with prior repertoire‐based studies in autoimmune disorders, we observed reduced clonotype richness (uCDR3 counts) and diminished diversity across multiple TCR and BCR chains in Ps, RA and UC. Such contraction of adaptive immune repertoires is typically interpreted as a consequence of chronic antigenic stimulation, clonal expansion and restricted immune diversity. Notably, although all three diseases exhibited repertoire contraction, the pattern of chain involvement differed, suggesting disease‐specific adaptive remodelling. For example, UC displayed reduced TCR diversity but relative expansion of BCR components, consistent with enhanced humoral involvement in intestinal inflammation. These findings reinforce the concept that autoimmune diseases share convergent features of clonal restriction while retaining disease‐specific immunological architectures.

‘ZHENG’, or TCM syndrome, is a unique diagnostic concept in TCM. It represents the core of TCM theory and guides its therapeutic approach. Dampness ZHENG, one of the most common TCM syndromes [47], has been associated with various autoimmune diseases. When dampness assails the body, it produces a graded spectrum of disorders that move inward from the periphery. On the outer layer, it induces erythematous scaling papules with associated itching or infiltration, reminiscent of psoriasis. Moving deeper, it affects the sinews and bones, causing rigidity, edema and arthralgia, as seen in rheumatoid arthritis. Internally, its stagnation in the bowels results in diarrhoea and the excretion of mucus, which is characteristic of ulcerative colitis. For instance, Wang et al. reported that Dampness‐heat blockage was the most prevalent ZHENG in patients with rheumatoid arthritis (RA), accounting for 39.4% of cases [25]. Similarly, Yang et al. found that Dampness‐heat blockage was the most common ZHENG in psoriasis, present in 35.1% of patients [48]. Additionally, Dampness‐heat syndrome is among the most frequent syndromes in ulcerative colitis (UC), representing 34.8% of UC cases [49, 50]. In this context, we do not restrict our analysis to any specific dampness‐centred complex pattern. Instead, drawing on our Dampness Pattern Scale, we prioritize the broader, more general features of the dampness pattern over specific sub‐types. These differences were reflected in clear separation on UMAP plots based on multiple variables. This suggests that autoimmune diseases with Dampness ZHENG may exhibit different immune patterns and responses compared to those without Dampness, supporting the need for differential treatment strategies based on TCM syndrome differentiation.

Although ZHENG is the foundation of TCM clinical practice, the traditional methods for diagnosing ZHENG are largely subjective and vary depending on the practitioner. Therefore, identifying objective biomarkers, especially through peripheral blood sampling, is crucial for the future advancement of TCM. In recent years, a growing number of studies have adopted multidisciplinary and interdisciplinary approaches to substantiate TCM diagnosis at the molecular level, thereby improving the precision and reproducibility of ZHENG differentiation. These efforts often employ omics‐based and multi‐omics strategies, such as proteomics and metabolomics [51]. In this study, we applied another omics‐based method, immune repertoire sequencing, to explore novel biomarkers for the differentiation of Dampness ZHENG.

Substantial research has demonstrated the diagnostic utility of TCR and BCR repertoire features in various diseases, including infections, autoimmune disorders and cancers [27, 52, 53]. These diagnostic signatures often involve the preferential use of specific V genes or V‐J gene combinations, as well as distinct CDR3 clonal types. In our study, we observed preferential V gene and V‐J gene usage, alongside differentially expressed CDR3 clones, when comparing patients with Dampness ZHENG to those with Non‐Dampness ZHENG. These results underscore the promise of immune repertoire sequencing as a method for identifying potential biomarkers to support TCM syndrome (ZHENG) differentiation.

With the aim of developing a clinically applicable biomarker for Dampness ZHENG, we first performed feature selection using LASSO regression, GLM and OPLS‐DA analyses [54, 55]. The intersection of these approaches identified four candidate V genes. These candidates were subsequently ranked according to their individual discriminative performance and the three top‐performing genes (*TRAV20*, *TRAV38‐2DV8* and *TRBV5‐1*) were selected for model development. We then compared the predictive performance of several classification algorithms, including boosted trees, decision trees, logistic regression, k‐nearest neighbours and random forest models. Logistic regression demonstrated the best overall performance and was therefore chosen as the final modelling approach. The resulting three‐gene signature exhibited favourable predictive performance in both the testing and validation cohorts. Moreover, independent validation is crucial for establishing the reproducibility and robustness of a biomarker, thereby facilitating its clinical translation. We validated our 3‐gene diagnostic model in an independent cohort (57 Dampness, 26 non‐Dampness), which yielded an accuracy of 74.7% and an AUC of 0.804 by using Logistic Regression. This confirms the model's potential for clinical diagnosis of Dampness ZHENG.

*TRAV20* has been structurally and functionally implicated in autoimmune celiac disease. In this context, TRAV20^+^ T cell receptors (TCRs) recognize deamidated gliadin peptides presented by HLA‐DQ8, with the gene contributing germline‐encoded CDR1α contacts that help shape antigen specificity [56]. *TRAV38‐2DV8*, identified as a cuproptosis‐related immune gene, is significantly associated with immunotherapy response and prognosis in lung adenocarcinoma (LUAD) [57]. Additionally, CD4^+^ mucosal‐associated invariant T (MAIT) cells exhibit a highly diverse TCR repertoire, marked by the specific usage of genes such as *TRAV8‐3*, *TRAV21* and other *TRAV8/12* family members, as well as highly variable CDR3α and TRBV sequences [58]. Interestingly, emerging immunogenomic and metabolomic evidence suggests that Dampness ZHENG constitutes a systemic immune imbalance, which predisposes affected individuals to impaired antiviral responses and heightened autoimmune activity [51]. Therefore, our findings may open a new avenue for investigating the molecular and immunological mechanisms underlying Dampness ZHENG, although further studies are warranted.

Several limitations should be acknowledged. The sample size, particularly within disease subgroups, limits statistical power and may constrain detection of subtler repertoire features [59, 60]. Furthermore, due to difficulties in collecting clinical samples from patients with ulcerative colitis, the external validation cohort also exhibits an uneven distribution across disease and dampness pattern categories, which may affect the validity of the validation. Larger, multi‐centre cohorts are required to validate and generalize these findings. Second, the absence of HLA genotyping precludes mechanistic linkage between V gene bias and antigen specificity. Third, functional validation of identified clonotypes was not performed. Further studies are needed to elucidate the pathogenic role of these differential clonotypes associated with Dampness ZHENG. Integration of single‐cell sequencing, antigen stimulation assays and microbiome profiling may further clarify the biological basis of Dampness‐associated immune signatures.

Despite these limitations, this study identifies Dampness Zheng—associated immune repertoire differences, offering novel insights into the molecular‐level objective characterization of TCM syndromes.

## 5 Conclusion

In summary, patients with psoriasis, rheumatoid arthritis and ulcerative colitis exhibit disease‐specific yet convergent adaptive immune repertoire contraction. Stratification by Dampness ZHENG reveals distinct clonal architectures and V gene usage patterns that transcend Western disease categories. A three‐gene TCR V‐segment model demonstrates promising diagnostic performance for distinguishing Dampness from non‐Dampness patients. These findings indicate that immune repertoire sequencing enables objective characterization of TCM syndromes through the distinct parameters of CDR3 abundance, repertoire diversity and V gene rearrangement. This work thus may pave the way for improved TCM‐based patient classification and the integration of Chinese and Western therapeutic strategies in clinical practice.

## Author Contributions

All listed authors contributed substantially to this work and approved its publication. Zhe Wang, Runyue Huang, Guangjuan Zheng and Chuanjian Lu designed the study. Lipeng Tang, Maojie Wang, Yuhong Yan and Shumin Qin contributed to participants' recruitment, sample collection, performed the clinical data analysis, and drafted the manuscript. Youbang Liang, Danni Yao, Liyan Mei, Hao Deng, Shuyan Ye, Xiumin Chen and Kaixin Gao contributed to participants' recruitment, sample collection and performed the clinical data analysis. Xirun ZHENG, Taohua Liu and Xinmin Qiu performed the TCR and BCR immune repertoire sequencing. Song Li, Xinjie Yu and Wenjing Pan analysed the TCR and BCR immune repertoire sequencing raw data. Shaogang Huang contributed to participants' recruitment and sample collection. Project funding was provided by Lipeng Tang, Runyue Huang and Chuanjian Lu. All authors have read and agreed to the publication of the work in this manuscript.

## Funding

This work was supported by the National Natural Science Foundation of China (Nos. U20A20397 and U23A6012), the Specific Fund of State Key Laboratory of Dampness Syndrome of Chinese Medicine (SZ2021ZZ23, SZ2021ZZ45, SZ2021ZZ3 and SZ2021ZZ37), Science and Technology Planning Project of Guangdong Province (2020B1111100005 and 2020B1111100006), Guangzhou Science and Technology Planning Project (No. 202206080006), Innovation Team and Talents Cultivation Program of National Administration of Traditional Chinese Medicine (No. ZYYCXTD‐C‐202204), National Traditional Chinese Medicine Leading Talents Support Program‐Qihuang Scholar [No. (2018)284], The Specific Fund of ‘Young Talents Program’ of Guangdong College of Traditional Chinese Medicine (SZ2022QN10), Hunan Provincial Natural Science Foundation of China (No. 2023JJ50199), Science Research Project of the Education Department of Hunan Province (No. 22A0385).

## Ethics Statement

This current study was approved by the Ethical Committee of Guangdong Provincial Hospital of Chinese Medicine (No. BF2020‐075‐01), and all associated procedures were carried out in accordance with approved guidelines. All participants signed an informed consent form.

## Consent

The authors have nothing to report.

## Conflicts of Interest

The authors declare no conflicts of interest.

## Supporting information


**Figure S1:** The intra‐group point distances and the inter‐group Euclidean distances among the Dampness and Non‐Dampness groups in psoriasis, rheumatoid arthritis and ulcerative colitis group.
**Figure S2:** Immunohistochemistry feature of Dampness and Non‐Dampness of Ps and UC tissue lesion. (A) Representative image of CD3 for PS tissue lesion for Dampness Ps and non‐Dampness Ps patients. (B) The density of CD3 positive cells in PS tissue lesion for Dampness Ps and non‐Dampness Ps patients. (C) Representative image of CD3 for UC tissue lesion for Dampness UC and non‐Dampness UC patients. (D) The density of CD3 positive cells in UC tissue lesion for Dampness UC and non‐Dampness UC patients. (C) Representative image of CD20 for UC tissue lesion for Dampness UC and non‐Dampness UC patients. (D) The density of CD20 positive cells in UC tissue lesion for Dampness UC and non‐Dampness UC patients. Mann–Whitney *U* test was used for B, D and F. Data represents the Mean ± S.D.

**Excel S1.** The training cohort‐Data Points.

**Excel S2.** An external validation cohort‐Data Points.

**Excel S3.** UMAP for Pso.

**Excel S4.** UMAP for RA.

**Excel S5.** UMAP for UC.

## Data Availability

The data that support the findings of this study are openly available in China National Center for Bioinformation/Beijing Institute at https://ngdc.cncb.ac.cn/gsa., reference number GSA: HRA013682.
