# Supplementary material for: Immune Repertoire Profiling Reveals Distinct Adaptive Immune Signatures of Dampness ZHENG Across Psoriasis, Rheumatoid Arthritis and Ulcerative Colitis
Source: Cell Prolif. 2026 Jul 18:e70265. Online ahead of print. doi: 10.1111/cpr.70265 (PMC13379743; doi:10.1111/cpr.70265)
Supplement: Supplementary file 1 — Figure S1: The intra‐group point distances and the inter‐group Euclidean distances among the Dampness and Non‐Dampness groups in psoriasis, rheumatoid arthritis and ulcerative colitis group. Figure S2: Immunohistochemistry feature of Dampness and Non‐Dampness of Ps and UC tissue lesion. (A) Representative image of CD3 for PS tissue lesion for Dampness Ps and non‐Dampness Ps patients. (B) The density of CD3 positive cells in PS tissue lesion for Dampness Ps and non‐Dampness Ps patients. (C) Representative image of CD3 for UC tissue lesion for Dampness UC and non‐Dampness UC patients. (D) The density of CD3 positive cells in UC tissue lesion for Dampness UC and non‐Dampness UC patients. (C) Representative image of CD20 for UC tissue lesion for Dampness UC and non‐Dampness UC patients. (D) The density of CD20 positive cells in UC tissue lesion for Dampness UC and non‐Dampness UC patients. Mann–Whitney U test was used for B, D and F. Data represents the Mean ± S.D. [file CPR-9999-e70265-s004.docx]

**Supplementary Files**

**
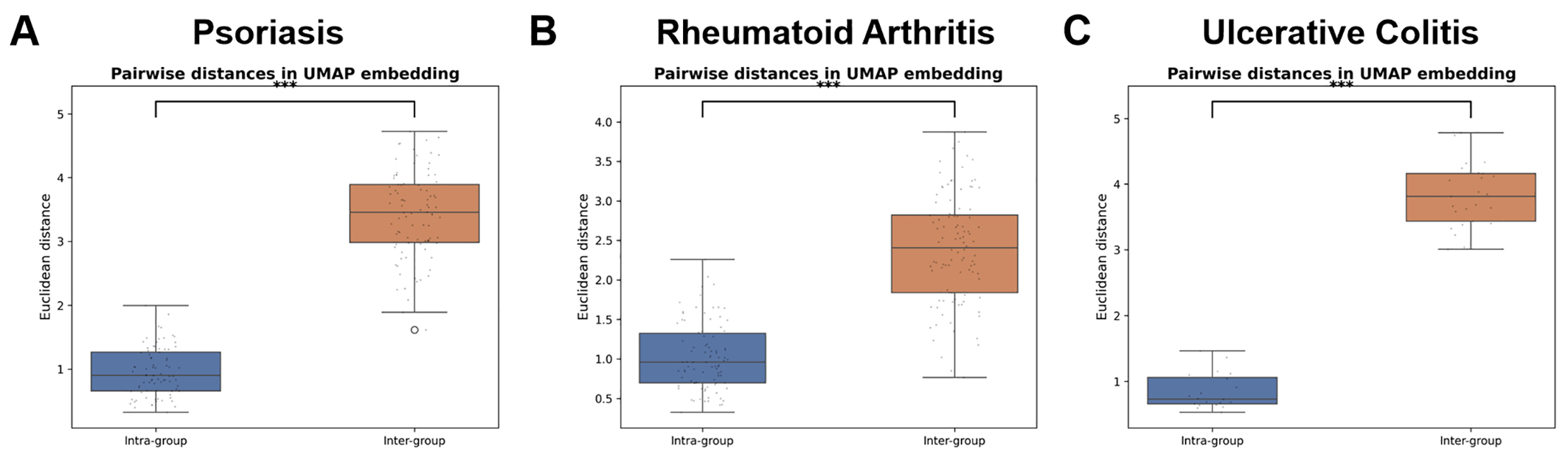
**

**Supplementary Figure 1. The intra-group point distances and the inter-group Euclidean distances among the Dampness and Non-Dampness groups in psoriasis, rheumatoid arthritis, and ulcerative colitis group.**


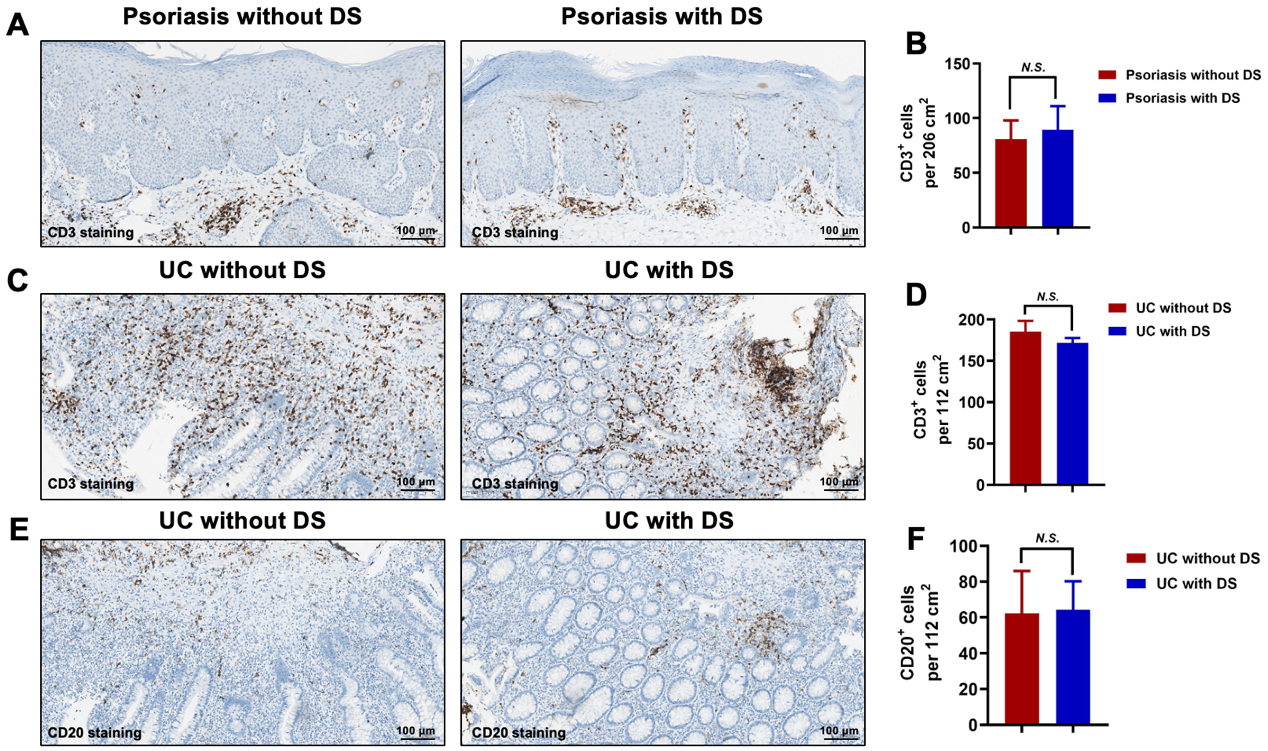


**Supplementary Figure 2. Immunohistochemistry feature of Dampness and Non-Dampness of Ps and UC tissue lesion.** (A) Representative image of CD3 for PS tissue lesion for Dampness Ps and non-Dampness Ps patients. (B) The density of CD3 positive cells in PS tissue lesion for Dampness Ps and non-Dampness Ps patients. (C) Representative image of CD3 for UC tissue lesion for Dampness UC and non-Dampness UC patients. (D) The density of CD3 positive cells in UC tissue lesion for Dampness UC and non-Dampness UC patients. (C) Representative image of CD20 for UC tissue lesion for Dampness UC and non-Dampness UC patients. (D) The density of CD20 positive cells in UC tissue lesion for Dampness UC and non-Dampness UC patients. Mann-Whitney *U* test was used for B, D and F. Data represents the Mean ± S.D.
